# Supplementary material for: Route selection in non-Euclidean virtual environments
Source: PLoS One. 2021 Apr 20;16(4):e0247818. doi: 10.1371/journal.pone.0247818 (PMC8057603; doi:10.1371/journal.pone.0247818)

Supplementary Material, Muryy and Glennerster

S1 Fig. ***Schematics of the labyrinths for Layout 1 (A,B,C) and Layout 2 (D,E,F).*** *A) 'Fixed' condition. B) One-wormhole condition; green target is inside wormhole W1. C) Three-wormhole condition; red, green and yellow targets are inside wormholes. The general layout (containing Start, which is marked as 'S', described as the 'ground floor' in the text) remained constant between conditions. The wormholes are marked with letters W surrounded by red and blue triggers. As the participant crossed a trigger, the environment changed without the participant being able to detect this transition, leading to the changes shown in the sub-schematics. Inside a wormhole, the participant could only walk along the route marked by the black dashed line. There were no junctions inside wormholes. D), E), F) show the same for Layout 2. Also see movies for A), B) and C).*

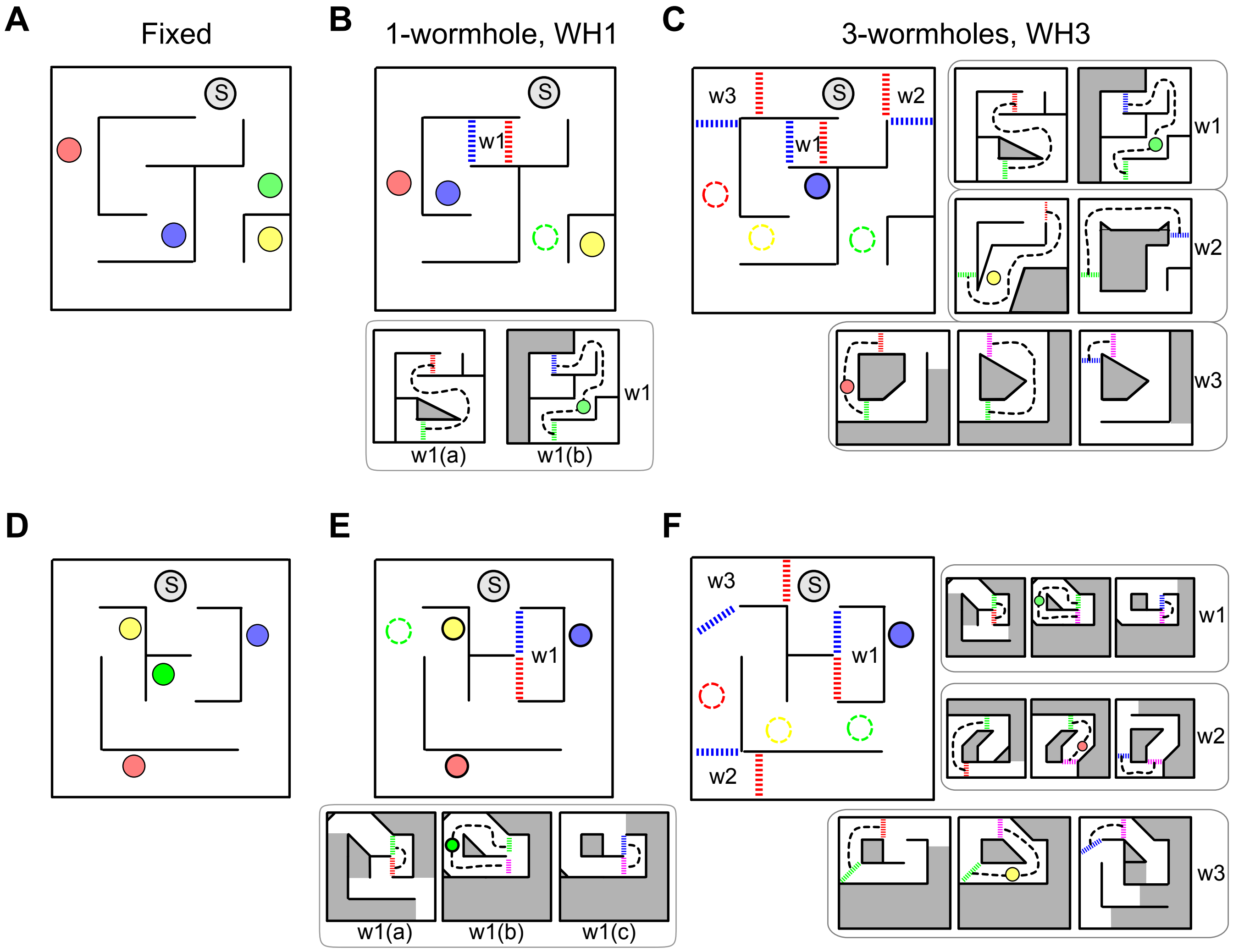

Supplement: S1 Fig — Schematics of the labyrinths for Layout 1 (A, B, C) and Layout 2 (D, E, F). A) ‘Fixed’ condition. B) One-wormhole condition; green target is inside wormhole W1. C) Three-wormhole condition; red, green and yellow targets are inside wormholes. The general layout (containing Start, which is marked as ‘S’, described as the ‘ground floor’ in the text) remained constant between conditions. The wormholes are marked with letters W surrounded by red and blue triggers. As the participant crossed a trigger, the environment changed without the participant being able to detect this transition, leading to the changes shown in the sub-schematics. Inside a wormhole, the participant could only walk along the route marked by the black dashed line. There were no junctions inside wormholes. D), E), F) show the same for Layout 2. Also see movies for A), B) and C). (PDF) [file pone.0247818.s001.pdf]
