# Supplementary material for: Route selection in non-Euclidean virtual environments
Source: PLoS One. 2021 Apr 20;16(4):e0247818. doi: 10.1371/journal.pone.0247818 (PMC8057603; doi:10.1371/journal.pone.0247818)
Supplement: S2 Fig — Bars show mean distances (in metres) travelled by all participants (n = 14) in each condition. Error bars indicate standard deviations. Horizontal black lines indicate lengths of the shortest solution, measured along the middle of the corridors. During the 5 rounds of the learning phase, the task was always the same (go from Start to Red-Green-Blue-Yellow). During the test phase (last 3 rounds), participants were asked to solve novel tasks, i.e. the routes were different on every test round. (PDF) [file pone.0247818.s002.pdf]

S2 Fig. ***Travelled distance per round.*** *Bars show mean distances (in metres) travelled by all participants (n=14) in each condition. Error bars indicate standard deviations. Horizontal black lines indicate lengths of the shortest solution, measured along the middle of the corridors. During the 5 rounds of the learning phase, the task was always the same (go from Start to Red-Green-Blue-Yellow). During the test phase (last 3 rounds), participants were asked to solve novel tasks, i.e. the routes were different on every test round.*

Layout 1

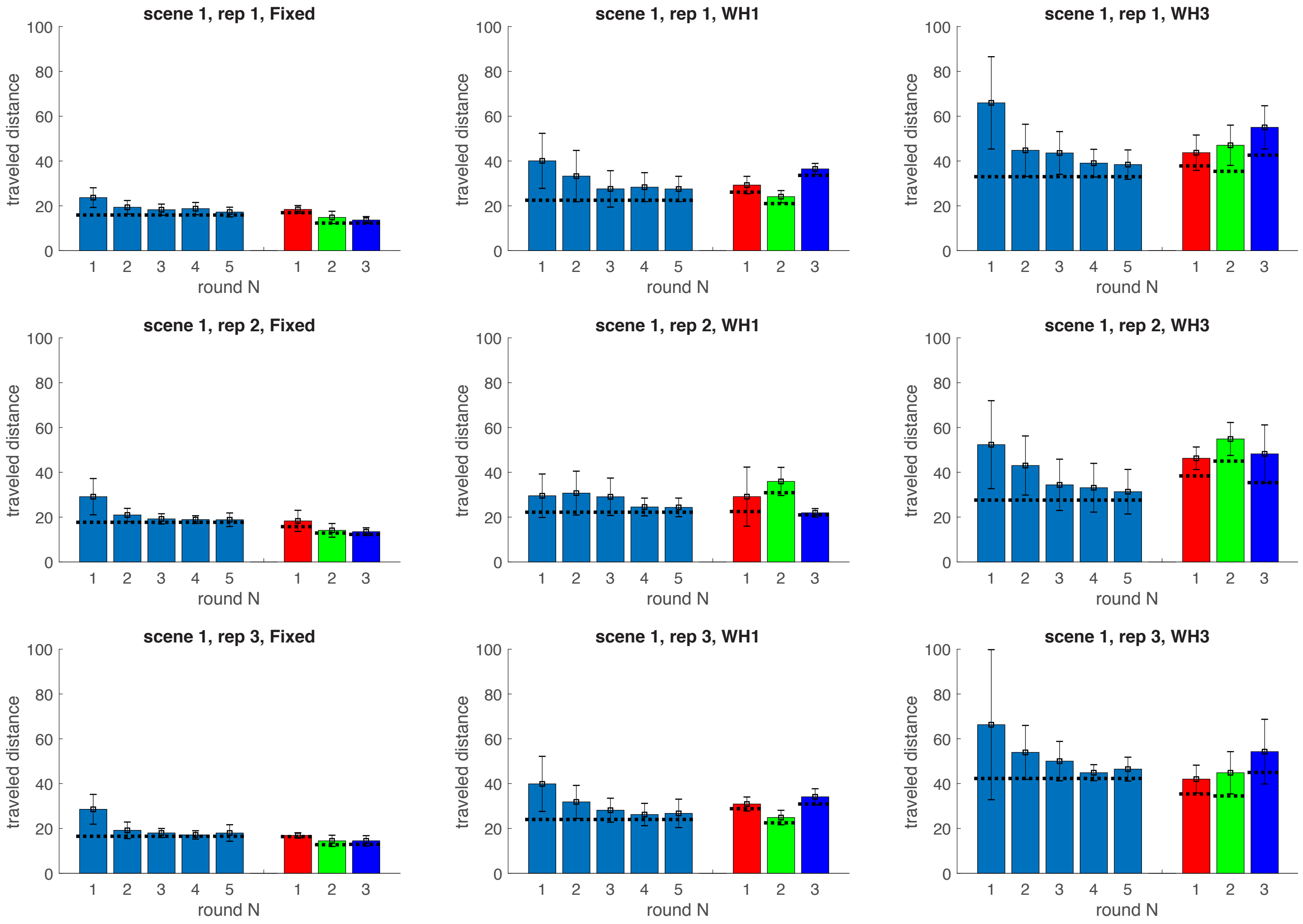

Layout 2

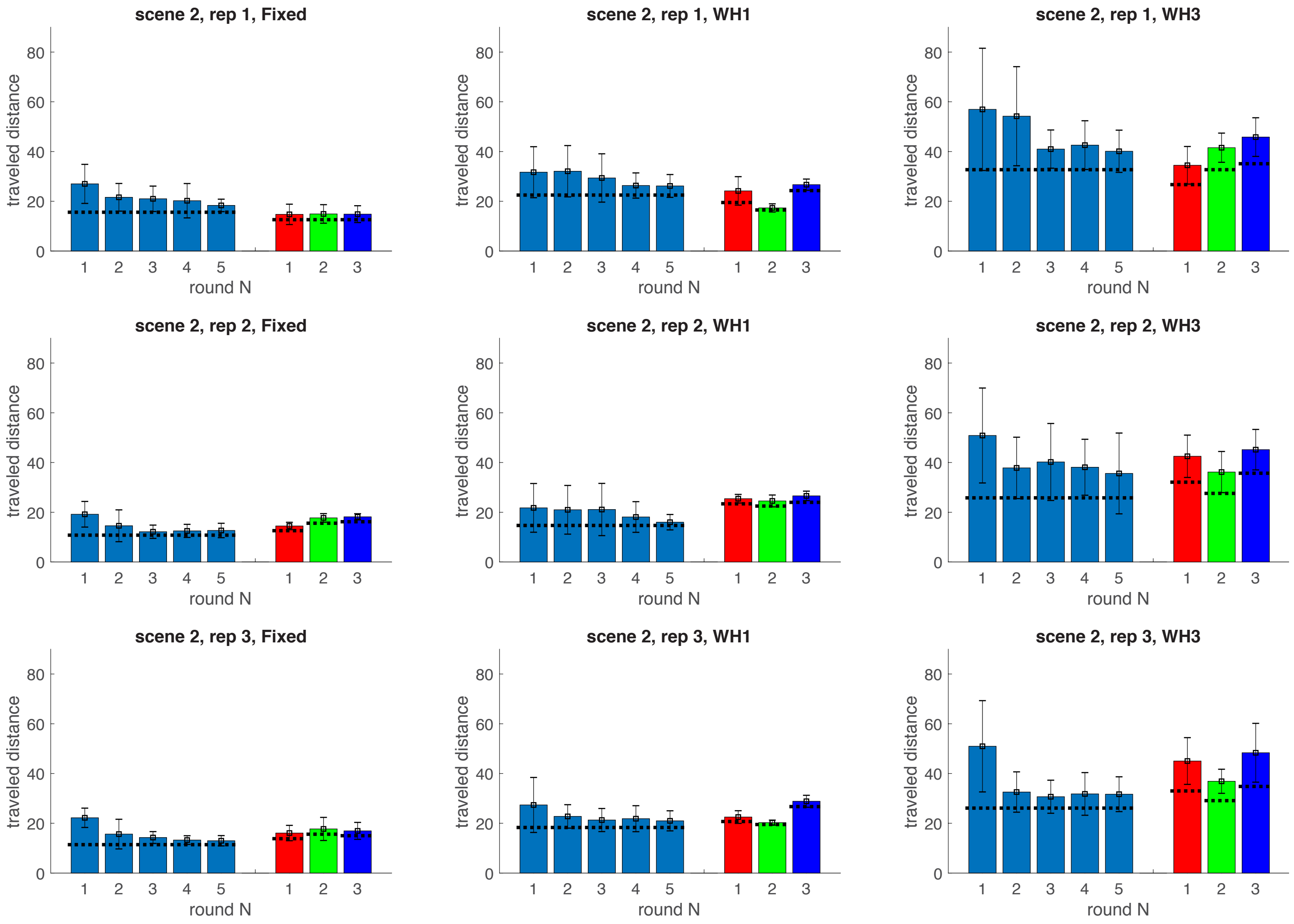

scene 2, rep 1, Fixed
scene 2, rep 1, WH1
scene 2, rep 1, WH3
scene 2, rep 2, Fixed
scene 2, rep 2, WH1
scene 2, rep 2, WH3
scene 2, rep 3, Fixed
scene 2, rep 3, WH1
scene 2, rep 3, WH3
traveled distance
round N
0
20
40
60
80
1
2
3
4
5
