# Supplementary material for: Route selection in non-Euclidean virtual environments
Source: PLoS One. 2021 Apr 20;16(4):e0247818. doi: 10.1371/journal.pone.0247818 (PMC8057603; doi:10.1371/journal.pone.0247818)

Supplementary Material, Muryy and Glennerster.

S3 Fig. ***Sketches drawn by participants right after experimental session.*** *The ground-truth schematics for both scenes in all conditions are shown in Fig. S1.*

**Original drawing**	**Reoriented to match Fig2**

Scene1, Metric, rep1, Participant8

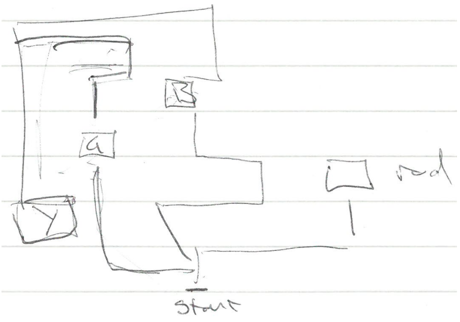

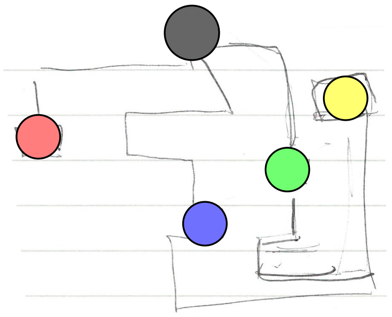

Scene1, WH1, rep1, Participant3

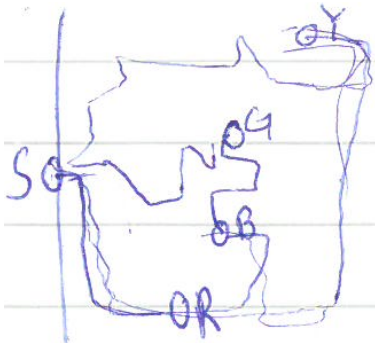

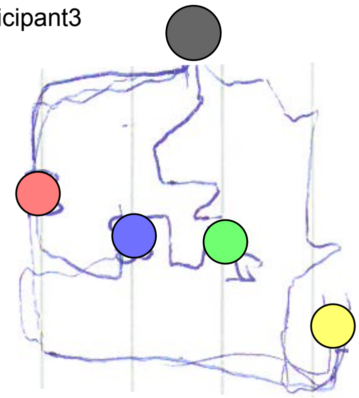

Scene1, WH1, rep2, Participant4

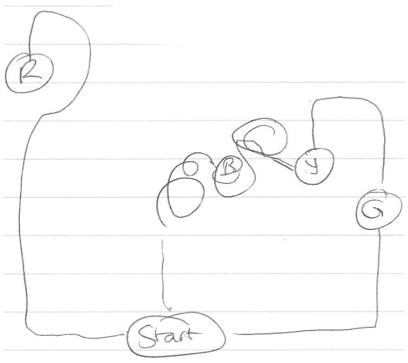

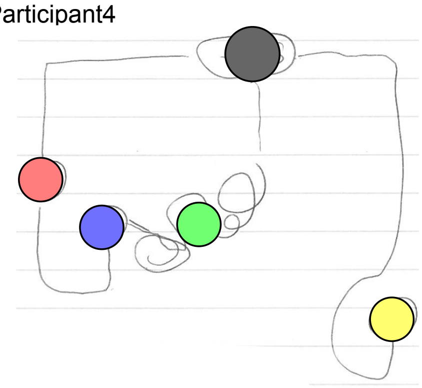

Original drawing **Reoriented to match Fig2**

Scene1, WH1, rep2, Participant7

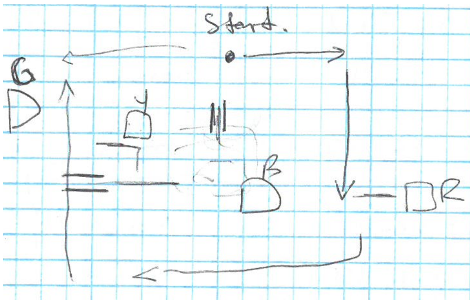

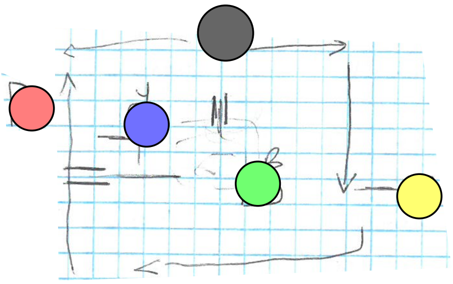

Scene1, WH3, rep1, Participant8

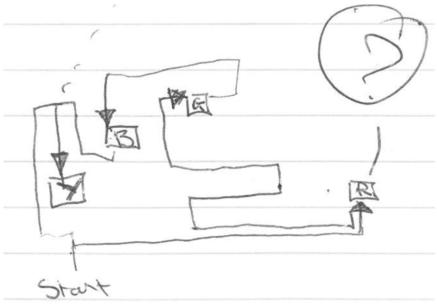

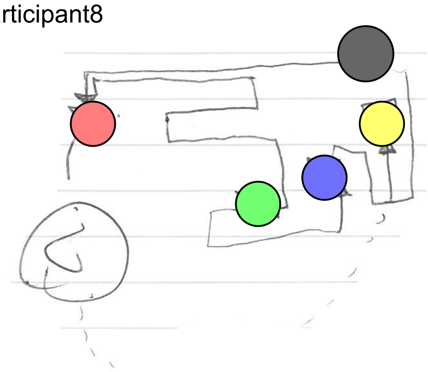

Scene1, WH3, rep2, Participant1

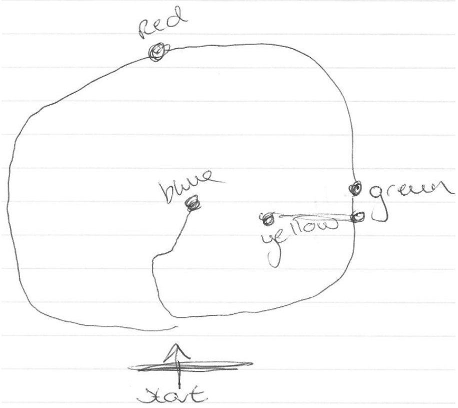

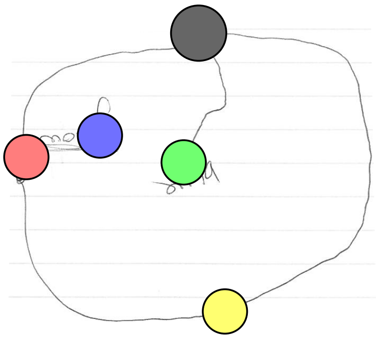

Original drawing **Reoriented to match Fig2**

Scene2, M, rep1, Participant8

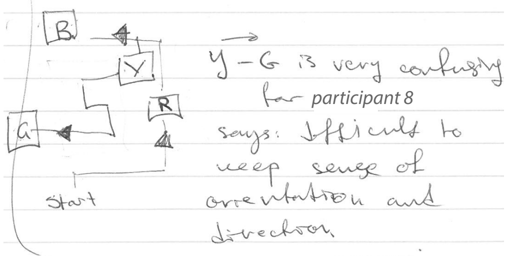

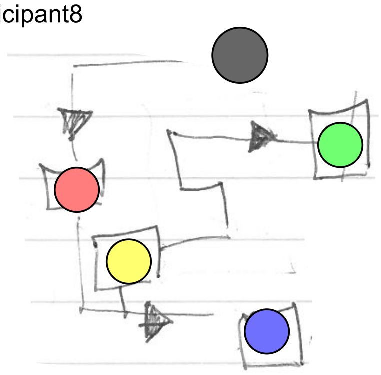

Scene2, M, rep2, Participant6

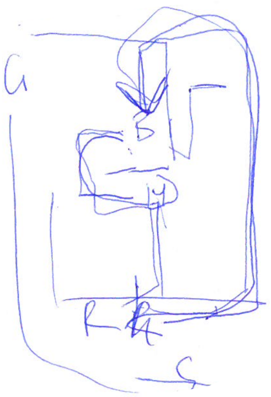

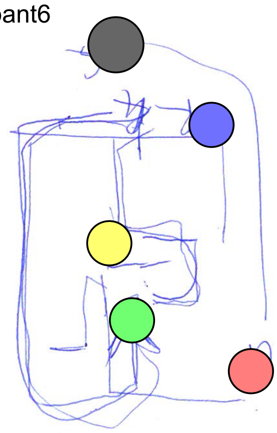

Scene2, WH1, rep1, Participant8

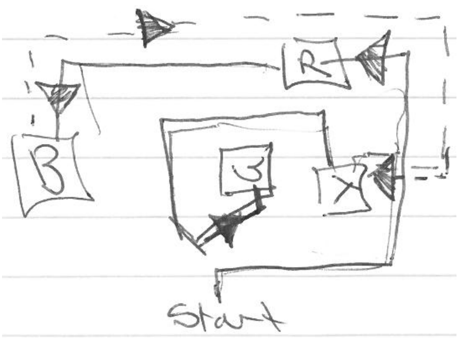

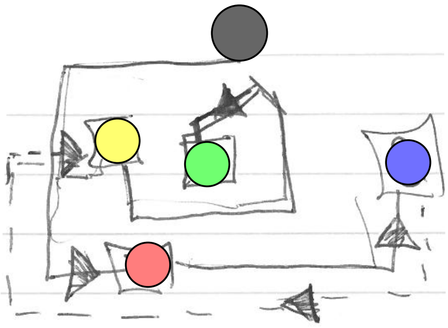

Original drawing **Reoriented to match Fig2**

Scene2, WH1, rep2, Participant4

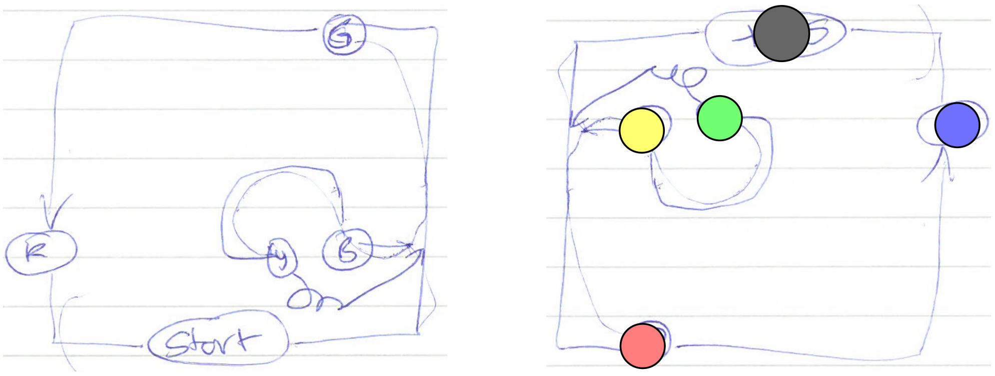

Scene2, WH1, rep2, Participant7

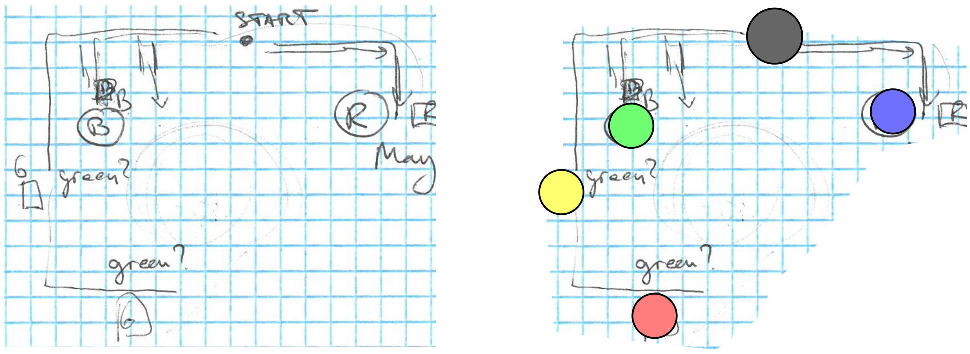

Scene2, WH3, rep1, Participant4

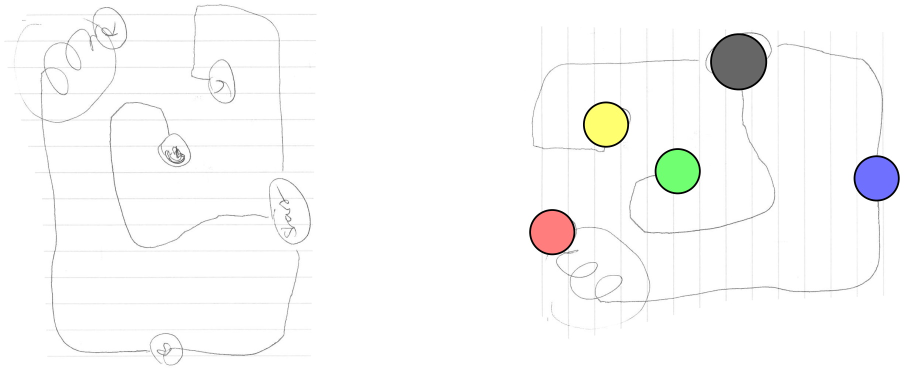

Scene2, WH3, rep2, Participant4

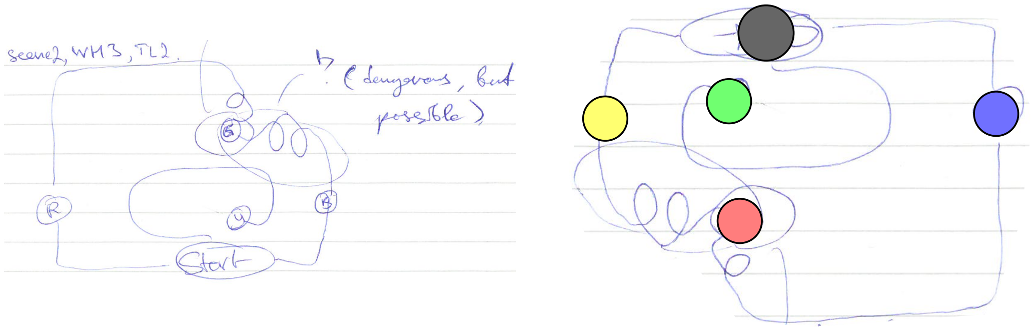

Supplement: S3 Fig — The ground-truth schematics for both scenes in all conditions are shown in S1 Fig. (PDF) [file pone.0247818.s003.pdf]
