# Supplementary material for: Route selection in non-Euclidean virtual environments
Source: PLoS One. 2021 Apr 20;16(4):e0247818. doi: 10.1371/journal.pone.0247818 (PMC8057603; doi:10.1371/journal.pone.0247818)

Supplementary Material, Muryy and Glennerster.

S4 Fig. ***Ability to point accurately against ability to find the shortest path.*** *The x-axis shows a measure of the ability of participants to find shorter paths: it is a ratio of travelled distance during a full round to the shortest distance of that round. The y-axis shows the ability of participants to point accurately (from Muryy and Glennerster (2018)): this is a mean pointing error (degrees) per round (mean over 8 pointings, since at the end of a round participants pointed 8 times). Solid lines show fitted linear regression models.*

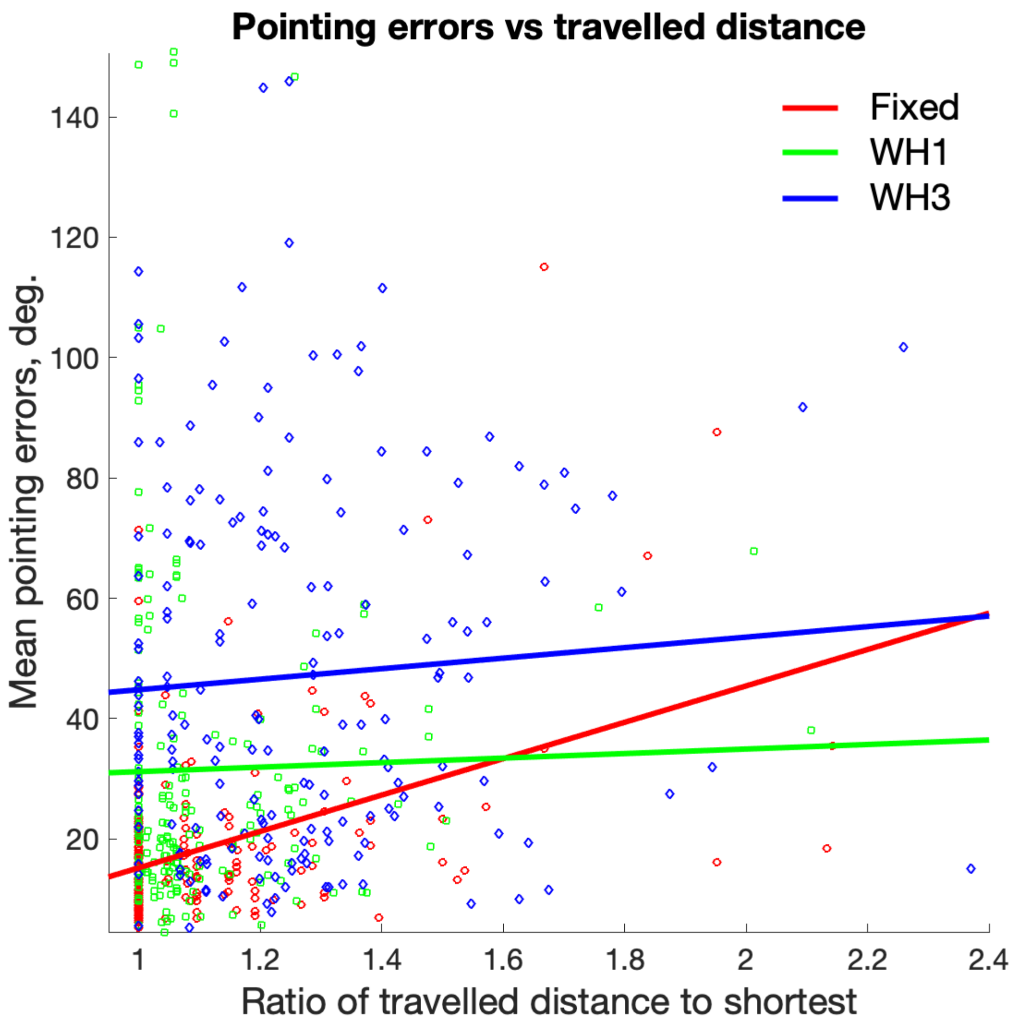

Supplement: S4 Fig — The x-axis shows a measure of the ability of participants to find shorter paths: it is a ratio of travelled distance during a full round to the shortest distance of that round. The y-axis shows the ability of participants to point accurately (from Muryy and Glennerster (2018)): this is a mean pointing error (degrees) per round (mean over 8 pointings, since at the end of a round participants pointed 8 times). Solid lines show fitted linear regression models. (PDF) [file pone.0247818.s004.pdf]
