# Supplementary material for: Route selection in non-Euclidean virtual environments
Source: PLoS One. 2021 Apr 20;16(4):e0247818. doi: 10.1371/journal.pone.0247818 (PMC8057603; doi:10.1371/journal.pone.0247818)
Supplement: S1 Data — Data and code to reproduce S2 Fig, ie distance travelled in all conditions by all participants. (ZIP) [file pone.0247818.s009.zip › muryy_glennerster_raw_data_fig_example/FigS2_scene2.pdf]

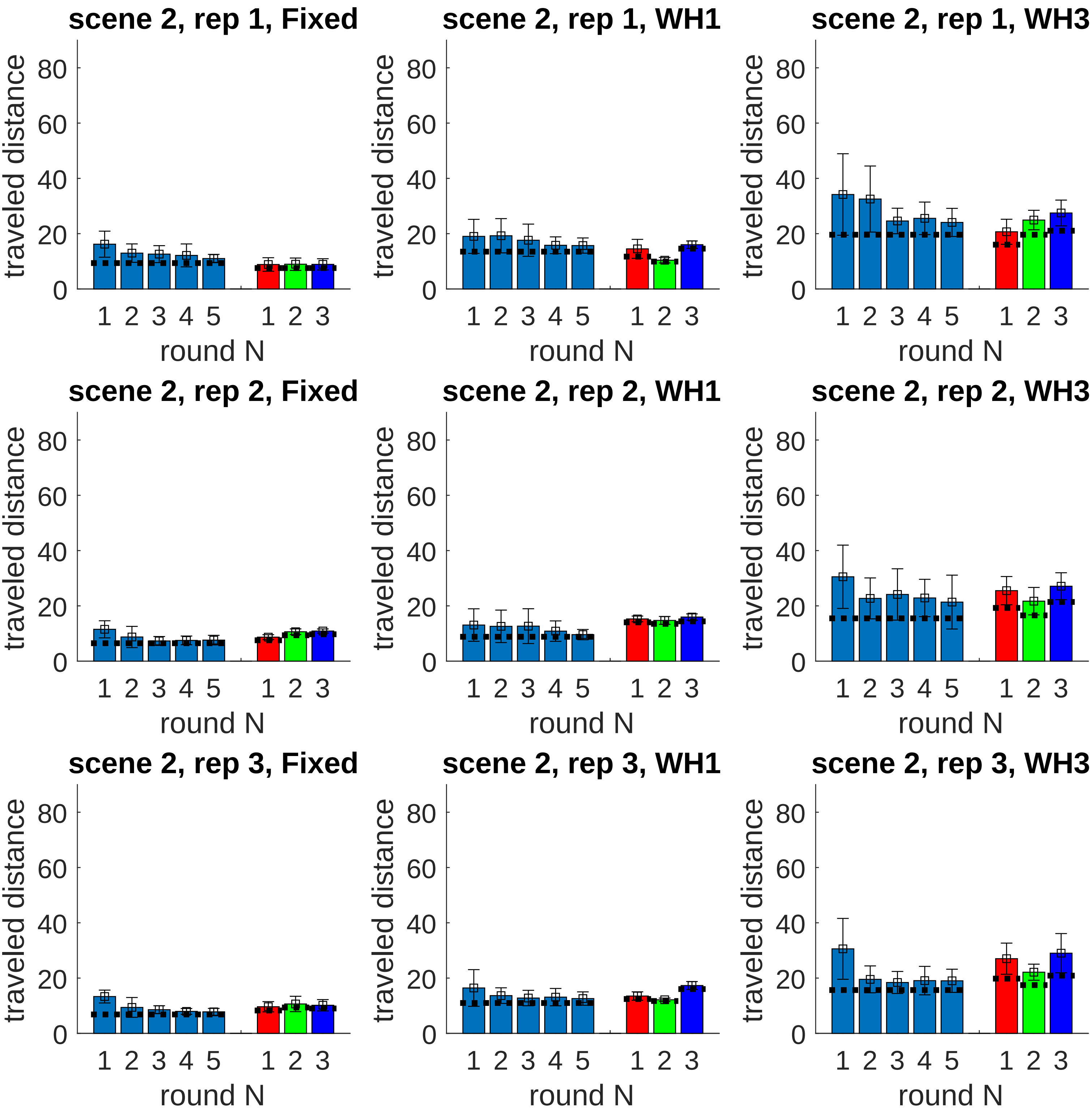
scene 2, rep 1, Fixed
scene 2, rep 1, WH1
scene 2, rep 1, WH3
scene 2, rep 2, Fixed
scene 2, rep 2, WH1
scene 2, rep 2, WH3
scene 2, rep 3, Fixed
scene 2, rep 3, WH1
scene 2, rep 3, WH3
traveled distance
0
20
40
60
80
1 2 3 4 5 1 2 3
round N
