# Supplementary figures and images for: Route selection in non-Euclidean virtual environments
Source: PLoS One. 2021 Apr 20;16(4):e0247818. doi: 10.1371/journal.pone.0247818 (PMC8057603; doi:10.1371/journal.pone.0247818)

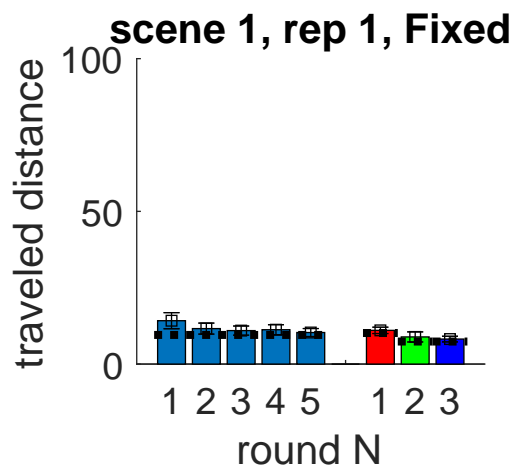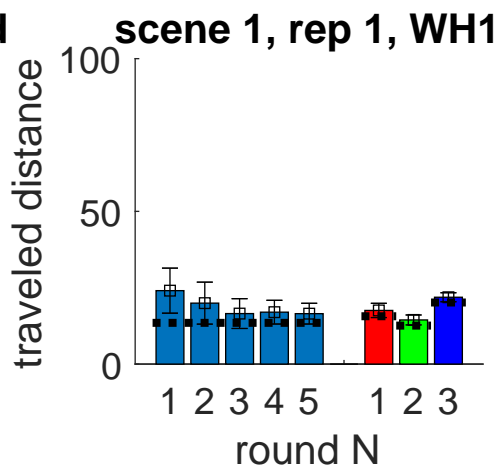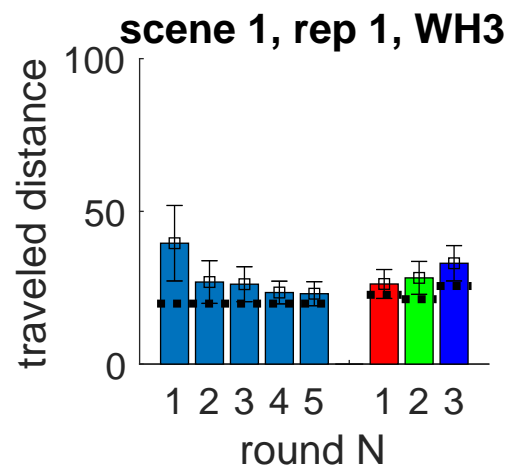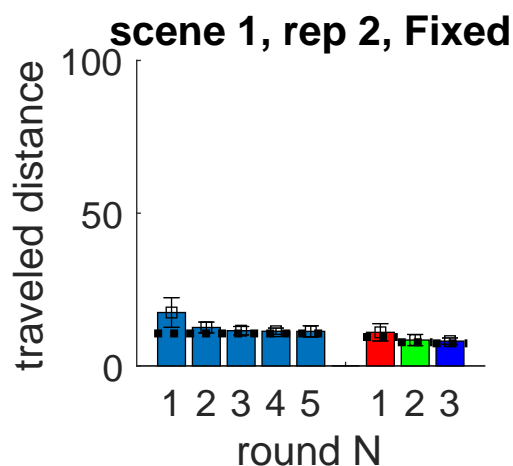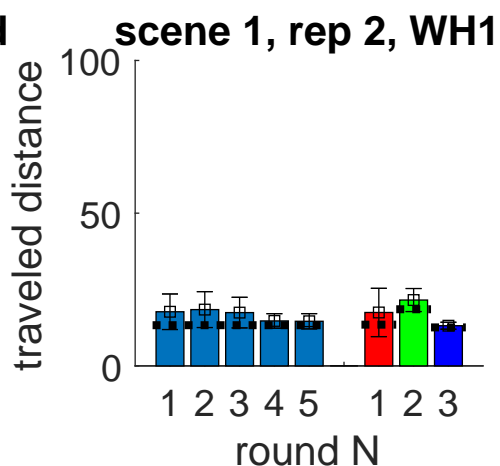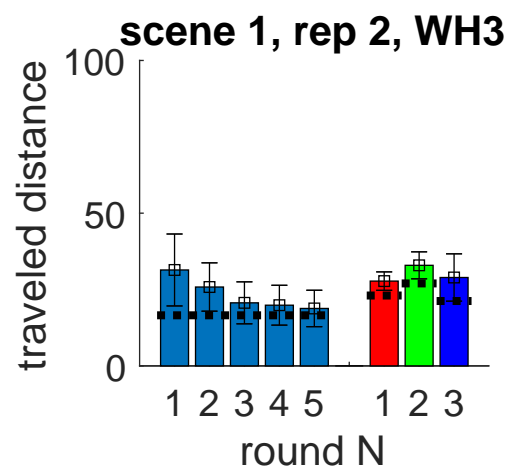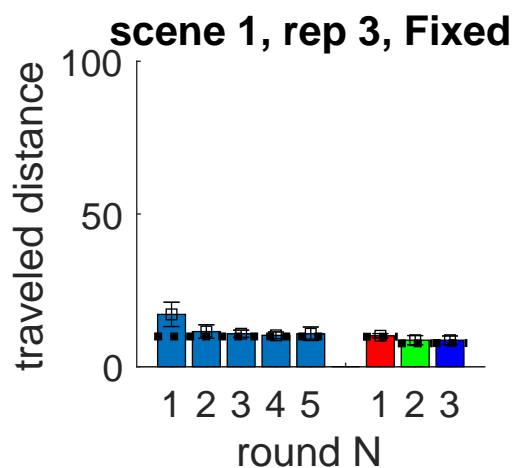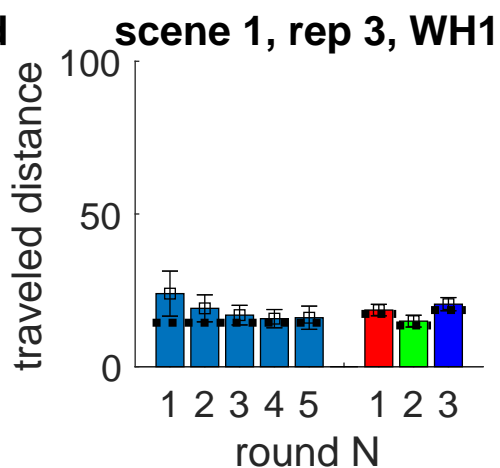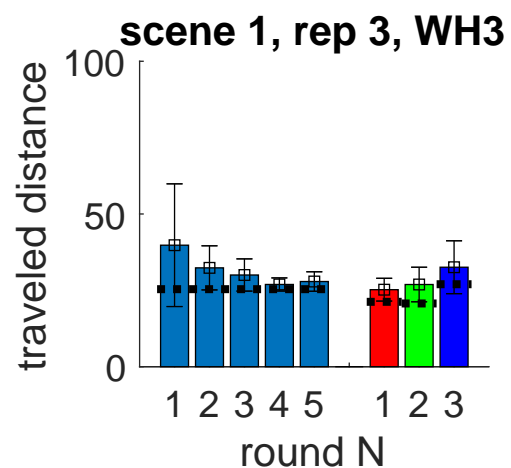

Supplement: S1 Data — Data and code to reproduce S2 Fig, ie distance travelled in all conditions by all participants. (ZIP) [file pone.0247818.s009.zip › muryy_glennerster_raw_data_fig_example/FigS2_scene1.pdf]
