# Supplementary material for: Route selection in non-Euclidean virtual environments
Source: PLoS One. 2021 Apr 20;16(4):e0247818. doi: 10.1371/journal.pone.0247818 (PMC8057603; doi:10.1371/journal.pone.0247818)
Supplement: Attachment — Submitted filename: muryy_glennerster_reply_dec2020.docx [file pone.0247818.s010.docx]

PONE-D-20-24724. Muryy and Glennerster

**Editorial comments**

*1. Please ensure that your manuscript meets PLOS ONE's style requirements, including those for file naming. The PLOS ONE style templates can be found at*

[*https://journals.plos.org/plosone/s/file?id=wjVg/PLOSOne_formatting_sample_main_body.pdf*](https://journals.plos.org/plosone/s/file?id=wjVg/PLOSOne_formatting_sample_main_body.pdf) *and* [*https://journals.plos.org/plosone/s/file?id=ba62/PLOSOne_formatting_sample_title_authors_affiliations.pdf*](https://journals.plos.org/plosone/s/file?id=ba62/PLOSOne_formatting_sample_title_authors_affiliations.pdf)

 Done

*2. We noted in your submission details that a portion of your manuscript may have been presented or published elsewhere.*

*"Figure 11 shows data from Muryy and Glennerster (2018)
Muryy A., Glennerster A. (2018) Pointing Errors in Non-metric Virtual Environments. In: Creem-Regehr S., Schöning J., Klippel A. (eds) Spatial Cognition XI. Spatial Cognition 2018. Lecture Notes in Computer Science, vol 11034. Springer, Cham.* [*https://doi.org/10.1007/978-3-319-96385-3_4*](https://doi.org/10.1007/978-3-319-96385-3_4) *The data are included for comparison with the current data. It is a re-plot of the data, not a copy of the figure."*

*Please clarify whether this publication was peer-reviewed and formally published. If this work was previously peer-reviewed and published, in the cover letter please provide the reason that this work does not constitute dual publication and should be included in the current manuscript.*

Yes, the Lecture Notes in Computer Science paper, Muryy and Glennerster (2018), is a peer-reviewed publication.

The previous Fig 11 was not reproduced from Muryy and Glennerster (2018) but it did re-plot some of the data shown in that paper in a way that makes it easy to compare with the data from the current paper. It is an important comparison we are making here (between the performance of participants on two different tasks in the same experiment). It is not dual publication because we do not claim that the data are newly published here, we are merely drawing the reader’s attention to the similarity between an aspect of the data in Muryy and Glennerster (2018) and the data in the current paper. This requires re-plotting the data to draw the link between the two and it helps the reader to have the two sets of data side by side to make the comparison.

*3. Your abstract cannot contain citations. Please only include citations in the body text of the manuscript, and ensure that they remain in ascending numerical order on first mention.*

Citation removed from the abstract.

***Reviewer #1:*** 

*My only (relatively small) critique is that the figures and data are quite complex. The authors do an admirable job breaking things down, but certain figures and their descriptions are difficult. For example, Figure 10 might be better broken down into multiple parts within each panel (a/b/c). If I’m understanding correctly, the authors are showing that on each sample the explanatory model that sample was drawn from was more likely to be the model it was drawn from? Seems logical enough, but I’m having a hard time tying in how the participant’s data maps onto that. It could be I’m missing something from the text, but these figures are quite busy and Fig. 10 in particular could be explained more clearly.*

Thank you for this. We have added text and an extra figure (Fig 11) to help explain the figure comparing the models in the current paper (Figure 12). Sampling from both models gives a rich picture of the extent to which the experimental data are a typical sample of each model, much more so than a likelihood ratio alone. A LR of 1 corresponds to the line of unity on these plots, and so a data point falling either side of this line favours one model or the other, but this method of plotting the data in the same space as samples from the opposing models provides more context about the extent to which the data are typical ‘draws’ from one or other model. We explain this in the paper (line 536 - Line numbers refer to the manuscript with track changes in the pdf).

*I think readers would also benefit from a figure or video illustrating how the two models operate. The shortest-distance model is clear; but what exactly the “rewarded-route” model is doing and even why it’s called the rewarded-route become easily lost in a paper that’s loaded with terms, abbreviations, etc.*

We have now added a figure to illustrate the step at which the probability of a given choice at a junction is updated after the route is ‘rewarded’ after the participant reaches the goal (Figure 8). After the learning phase is complete, these are the likelihoods used in the matrices for the rewarded-choice model as shown in Figure 9. *Two other minor points:
1. I did not see a discussion of sample size. How was 14 participants decided? I also see a high proportion of participants were excluded. Do the authors have any data on these subjects that might show them to be different (e.g., self-report, gender, etc.? )*

It was not possible to carry out a power calculation in advance to estimate the appropriate sample size as we could find no similar study comparing models of this type. We show the likelihood ratio of the two models for each individual participant (Fig 12) so the conclusion does not rest only on the values reported for the combined data.

In truth, we originally planned to test 12 participants but ended up testing two more. In case we might be accused of *p*-hacking, we have re-run the ANOVAs reported above with these two participants omitted. For the result we reported in the previous manuscript, the effect of condition on the relative likelihood of the models remains significant (F(2,35) = 12.4, p = 0.00004). The difference that reviewer 2 asked about, between one-wormhole and three-wormhole conditions, remains significant (F(1,23) = 7.69, p = 0.018) and but the difference between ‘Fixed’ and one-wormhole is no longer quite significant (F(1,23) = 4.60, p = 0.055). The difference between ‘Fixed’ and one-wormhole conditions is not a critical comparison in the paper. Instead, for both Muryy and Glennerster (2018) and the current paper, it is the trend from ‘Fixed’ to one-wormhole to three-wormholes that we emphasise.

The higher than usual rate of exclusions were likely to be due to the scaled scene which some potential participants found uncomfortable. We flag this in the text (line 150) and include the gender ratio of the excluded participants.

*2. I did not find the data available for this study anywhere in the manuscript or supplementary materials.*

We emailed KaytJane at PLoS ONE on 13^th^ September 2020 with the raw data (paths of all participants in all conditions) and example code that uses the raw data to reproduce figure 4. We said that this might be useful to reviewers. At the same time, we put the data on bioRxiv. We hope the PLoS ONE publication will also include a link (line 729).

<http://glennersterlab.com/muryy_glennerster/muryy_glennerster_data.zip>

and

<https://www.biorxiv.org/content/10.1101/2020.08.14.250621v2.supplementary-material>

***Reviewer #2****:  I do have some questions, both theoretical and methodological, about the results and their interpretation. I think the following comments need to be addressed before the paper can be accepted for publication.*

*1. The authors argue that the shift away from metric path to rewarded route depends on environmental complexity, but unfortunately complexity is confounded with Euclidean and non-Euclidean environments. It is thus critical to compare the goodness-of-fit for the two models between WH1 and WH2 (Figure 9A), which differ only in complexity. As far as I can see, they authors find a main effect of environment for the metric model fit, but do not make statistical comparisons between WH1 and WH2. These should be reported.*

We now report these. The ANOVA results for the shortest-distance model for ‘Fixed’ versus ‘one-wormhole’ is significant, as is the difference between ‘one-wormhole’ and ‘three-wormhole’ conditions, line 496 (Line numbers refer to the manuscript with track changes in the pdf). These results do not support the contention that there is a sudden jump in performance from ‘Fixed’ to non-Euclidean structure with no effect thereafter of adding more wormholes as one might expect if going from Euclidean to non-Euclidean environments was the crucial factor. We have changed the Discussion to emphasise more clearly than before that complexity and non-Euclidean structure co-vary in the experiment. We have also clarified the rationale for an experiment that could disentangle complexity and non-Euclidean structure experimentally if a larger virtual environment was available (lines 624-632).

*2. The authors seem to assume that rewarded-route learning yields knowledge of the “connectivity” of the environment. But they fail to distinguish between route knowledge (place-goal-action associations) and topological graph knowledge (enabling novel detours to the same goal, e.g. Chrastil & Warren, 2014). This difference should be described in the introduction (Lines 95-106) and acknowledged as an alternative in the Discussion (p. 22-23) – see next comment. Does a reinforcement learning algorithm only yield route knowledge, or could it produce topological graph knowledge – true connectivity?*

We now make this distinction clear both in the Introduction (line 107 - 113) and in the Discussion and refer to Chrastil & Warren, 2014. We have changed the term ‘rewarded route’ to ‘rewarded choice’ throughout because the model refers to a participant’s choice at an individual junction not the overall connectivity of the graph.

*3. The authors report a shift away from the metric path model, and toward the rewarded route model, with environmental complexity. But there is a third hypothesis: the shortest topological path (number of edges), which depends on connectivity but not edge weights. Can the present data distinguish a topological path model from the other two? What is the correlation between topological distance and metric distance in their mazes, and between topological distance and rewarded route?*

We have added a paragraph (line 633 - 642) citing Chrastil and Warren’s (2014) study comparing shortest topological and metric routes. Our wormholes keep the topology the same but change the metric distance of edges. We have not carried out a formal analysis, but we discuss this hypothesis as a potential account of the data.

*4. In the Introduction (Lines 91-92) the authors say that a labeled graph with precise and internally consistent local metric information “is indistinguishable in practice from a Euclidean map.” I don’t think this is correct. The difference is that the local information is not embedded in a global coordinate system, so the graph has nothing to say about the spatial regions in between its nodes and edges. Clever experimental tasks might be designed to probe knowledge of those regions.*

We have adjusted the text to reflect this distinction. We now make it clear that the claim we make about the inability to separate Euclidean representation experimentally from a highly calibrated labelled graph refers only to tasks involving objects or an observer at nodes in the graph (see Discussion, line 611).

*5. Lines 159-160: I’m concerned that eyeheight was only 1m above the ground plane. I understand that the entire environment scaled proportionally, but if actual eyeheight can be perceived (e.g. from binocular cues) then visual and idiothetic information for metric distances would be inconsistent, which may interfere with learning metric path lengths.*

Participants perceived themselves to be in a normal sized room, consistent with previous reports using similar scaling of an entire environment, i.e the eyeheight cue dominated over the scaling that could be provided by interocular separation or proprioception while walking. As the reviewer points out, there is a potential conflict here but, perceptually, it seems to be resolved in favour of the eyeheight cue. We now discuss this in the text (lines 464 - 472) and note that any effects of such conflict would be expected to have an equal effect on the ‘Fixed’, one-wormhole and three-wormhole conditions. *Details
• Line 16, Abstract: Is Muryy & Glennerster (2018) published?*

Yes, it was peer-reviewed and published in LNCS in 2018. Apologies, the full citation is now included.

*• Lines 135-6: “We called physically possible mazes ‘Fixed’, for short.” Did you use this term with the subjects, or are you just using it in the manuscript for the reader’s benefit? That is, were the subjects aware of which maze was ‘Fixed’ and which not?*

The task and instructions were identical for all three conditions. No, we did not use these terms with participants. All participants became aware that the three-wormhole condition was not a normal environment.

*• Line 153: What was the motion-to-photon latency of the HMD?*

We have published the motion-to-photon latency of our system using the nVis SX111 headset (40ms) and the method used. We now add a citation (line 162).

*• Lines 265-266: “For different repetitions of the same Layout, the structure of the labyrinth and target locations were identical, but the colours of the targets were changed.” If the target colors changed on every repetition, how could subjects ever learn the connectivity of the environment? Or were the target colors always the same in each Environment (Fixed, WH1, WH2)?*

Participants recognised corridors and junctions and grey boxes even if the colour of the target spheres changed between different repetitions. So, no, the target colours were not always the same in each environment. We have added some extra explanation (line 279 - 288) in the Methods to make this clear.

*• Lines 537-542: These two sentences seem inconsistent. The authors predict both that the rewarded-route model, and that the shortest-distance model, would be the best model when subjects are unable to judge between ‘Fixed’ and non-Euclidean environments.*

We have now added text to the Discussion (line 649 - 664) to clarify the two predictions. These depend on the information that is assumed to be present in the labelled graph (i.e. before or after information about the metric distance along each edge is stored in the graph).

*• On Lines 84-85 the authors say that graph structure and local metric information can be acquired in parallel, contrary to Siegel & White (1975), but consistent with Ishikawa & Montello (2006) and Warren (2019). Then on Lines 519-520 they say that observers start with connectivity and then add local metric information, consistent with Siegel & White (1975). This seems inconsistent, and their data do not support one or the other: some metric information could be acquired even in WH2.*

We have changed these sections to remove any potential ambiguity. We say, “the information about edges can become so accurate that tasks such as pointing from the current node in the graph to an object at another node can be as accurate as they would be given a Euclidean map, making it impossible to distinguish between a graph and a map for such tasks.” (line 114 - 116). We no longer include any comment about learning in parallel. Yes, local metric information about edges can be acquired in the three-wormhole condition and in the paper we draw attention to the fact that there are Euclidean parts of the wormhole mazes (eg line 771).
